# Knowledge, Attitude, and Practices on Water, Sanitation, and Hygiene among Rural Residents in Tigray Region, Northern Ethiopia

**DOI:** 10.1155/2020/5460168

**Published:** 2020-03-19

**Authors:** Abera Aregawi Berhe, Abraham Desta Aregay, Alemnesh Araya Abreha, Asfawosen Berhe Aregay, Ataklti Weldegebrial Gebretsadik, Degnesh Zigta Negash, Equbay Gebru Gebreegziabher, Kiros Ghebremedhin Demoz, Kiros Ajemu Fenta, Nega Bezabih Mamo

**Affiliations:** ^1^School of Public Health, College of Health Science, Mekelle University, Mekelle, Tigray, Ethiopia; ^2^Tigray Health Research Institute, Mekelle, Tigray, Ethiopia

## Abstract

**Background:**

Poor hygienic practices, inadequate water supply, and poor sanitary conditions play a major role in the spread of infectious diseases. Lack of knowledge, attitude, and practices (KAP) on WASH is one of the most imperative causes for transmission of infectious diseases. Therefore, the aim of this study was to assess knowledge, attitude, and practice of rural residents on water, sanitation, and hygiene in Tigray, Ethiopia.

**Methods:**

A community-based cross-sectional study was conducted from June to July 2018. Multistage cluster sampling technique was used to collect data from 759 households in Tigray region, Northern Ethiopia. A standardized questionnaire was used to collect data on knowledge, attitude, and practice on water, sanitation, and hygiene (WASH). Descriptive data analysis was done to present the study findings.

**Results:**

The response rate was 99.6%, and 574 (75.9%) of the respondents were females. Good knowledge, favorable attitude, and good practice on WASH were observed in 42.2% (95% CI: 38.7%, 45.7%), 48.5% (95% CI: 44.9%, 52.0%), and 49.2% (95% CI: 45.6%, 52.7%) of the respondents, respectively.

**Conclusions:**

Poor knowledge, unfavorable attitude, and poor practice on WASH were common amongst the residents in rural Tigray, Northern Ethiopia. Therefore, the health extension programs at primary health care should be revitalized in a way that can enhance the interventional measures to improve knowledge, attitude, and practice on WASH.

## 1. Introduction

The effects of poor water, sanitation, and hygiene (WASH) affect every aspect of health and development, hinder economic and social development, and constitute a major hurdle to poverty alleviation [1]. Many communicable diseases can be effectively managed by improving WASH practices. Waterborne disease prevalence can be reduced through implementing the three key WASH practices. Safe disposal of faeces and hand washing with soap at critical times can reduce prevalence of waterborne diseases by 30% and 40%, respectively. Likewise, safe treatment and storage of drinking water can reduce the prevalence of waterborne diseases by 30–50% [2].

Globally, 2.3 billion people lack basic sanitation (892 million people practiced open defecation), 844 million people lack basic drinking water, and 2.5 million people lack of improved sanitation [3]. In developing countries, WASH is one of the most important felt needs in public health in this 21^st^ century. However, about 842,000 people die as a result of inadequate WASH each year, representing 58% of the total diarrheal deaths [4]. In sub-Saharan Africa, WASH remains one of the major public health challenges with very low coverage [5]. Nearly, half of the population (319 million) does not use WASH facilities, 58% in sub-Saharan Africa lack basic drinking water, and only 15% have handwashing facilities with soap and water [3]. In Ethiopia, the 2016 Demographic and Health Survey (DHS) report indicated that only 57% the households (HHs) in rural areas obtain their drinking water from improved sources and 39% have no toilet facility. The prevalence of diarrhea episodes in the community was reported to be 12% [6].

Lack of knowledge, attitude, and practice (KAP) on WASH is one of the most imperative causes for transmission of infectious diseases [7, 8]. Effectiveness of WASH depends not only on the provision of WASH facilities but also, and most importantly, on the compliance of individuals. Unless people have adequate KAP in relation to WASH, mere access to the services is not sufficient to mitigate health problems related to unsafe water, poor sanitation, and hygiene [9]. The extent of safe WASH practices can be determined by the people's knowledge and attitudes towards WASH [10].

Tigray region has been implementing WASH projects such as One WASH and as a package of health extension programs, and it has been implemented since 2003 by considering its significance for the protection of public health and reducing WASH-related mortality and morbidity [11]. Despite continued efforts of WASH programs, frequent WASH-related epidemics including acute watery diarrhea (AWD) are still persistent with high proportions in the region. Therefore, understanding KAP on WASH in rural areas of Tigray, Northern Ethiopia, is essential to design and apply appropriate strategic measures to prevent WASH-related diseases. This study could also be used as a baseline to assess the impact of WASH interventions.

## 2. Materials and Methods

### 2.1. Study Design, Setting, and Sampling

**Figure 1:**
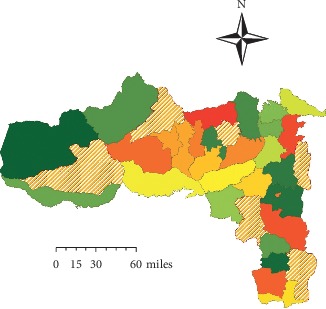
Map of Tigray region and selected districts shaded by radiation overlay color.

Community-based quantitative cross-sectional study design was used. Observation was embedded to the quantitative cross sectional of this study. The study was conducted from June to July 2018 in Tigray region, Northern Ethiopia (Figure 1).

Sample size was calculated using a single population proportion formula. Based on a 2011 survey in Tigray, latrine utilization was 34% [12]. Design effect of 2 was used as multistage sampling technique. The following assumptions were made to determine the minimum sample size:

- Latrine utilization of 34%, *P*=0.34.
- Two-sided standard *Z*-score *Z*_*α*/2_ = 1.96; corresponding to a 95% confidence level.
- Margin of error/relative precision *ε* = 0.05 (5%).
- Design effect *d* = 2, and contingency for nonresponse rate = 10%:

(1) $$\begin{array}{c} n=\frac{Z_{\alpha /2}^{2}p\left(1-p\right)}{\varepsilon^{2}}*d;\;Z_{\alpha /2}=1.96;\;\alpha =0.05;\;p=0.5\;\&\;1-p=0.5;\;\varepsilon =0.05,\\ n=\frac{{\left(1.96\right)}^{2}*0.5*0.5}{{\left(0.05\right)}^{2}}*2=345*2=\underline{690},\\ n_{f\;}=690+\left(0.1*690\right)=\;\underline{\underline{759}}\;\left(\text{total sample size}\right), \end{array}$$

where *n*_f_ is the final sample size.

In the 1^st^ stage of sampling, 6 districts from the region were randomly selected by a lottery method and the calculated sample was allocated to each selected district using probability proportional to size (PPS). In the 2^nd^ stage, 2 Kebelles (lowest administrative unit) were randomly selected from each selected district randomly by a lottery method, and a sample was allocated to each selected Kebelle using PPS. In the 3^rd^ stage, HHs were selected using a sampling interval (*K*) where every household at *K*/2 + 1 was selected for an interview and observation. In a compound with more than one HHs, only one HH head was included.

### 2.2. Operational Definitions

#### 2.2.1. Poor Knowledge

A mean knowledge score of ≤0.50 was considered as poor knowledge.

#### 2.2.2. Good Knowledge

A mean knowledge score of >0.50 was considered as good knowledge.

#### 2.2.3. Negative Attitude

A mean attitude score of ≤0.60 was considered as having negative attitude.

#### 2.2.4. Positive Attitude

A mean attitude score of >0.60 was considered as having positive attitude.

#### 2.2.5. Poor Practice

A mean practice score of ≤0.50 was considered as poor practice.

#### 2.2.6. Good Practice

A mean practice score of >0.50 were considered as good practice.

#### 2.2.7. Handwashing Facility

Any setup of a container with water and soap in the household compound for handwashing purpose observed at the time of data collection.

#### 2.2.8. Cleanliness

The household compound is free from solid and liquid wastes as decided by the data collector's observation.

### 2.3. Data Collection and Analysis

The study variables include sociodemographic characteristics and characteristics related to knowledge, attitude, and practice on water, sanitation, and hygiene. The study used an interviewer-administered structured questionnaire to collect data from the head of households or the oldest adult son/daughter in the absence of the head of households. Observational checklists were used to capture and ensure the practices on household compound cleanliness and latrine utilization (observing fresh feaces) in addition to the self-reported data. The questionnaire was adapted from different literatures considering the local situation. Data were collected by 6 trained and experienced environmental health professionals under the supervision of the principal investigators to ensure data quality. Data completeness and validity was monitored on a daily basis by the researchers, and the tools were developed in English and then translated to the local language Tigrigna.

The main variables were knowledge, attitude, and practice on WASH. In measuring those variables, knowledge-related questions (Table 1), attitude-related questions (Table 2), and practice-related questions (Table 3) on WASH were used in this study. For each question, correct response was given a score of 1, while a wrong response was given a score of 0. The scores were added, and the mean score was calculated. Similarly, attitude was measured using a Likert scale type (1–5). The attitude responses were as follows: very satisfied or strongly agree (scale 5) and agree or satisfied (scale 4) were considered as favorable attitudes on WASH. In measuring practice, each correct response was given a score of 1, while a wrong response was scored as 0.

Data were entered in Epi-data v.3.1 software, and they were analyzed using STATA V.14.1 statistical software. Data cleaning was done by running frequencies for each variable in STATA 14.1 to check outliers, inconsistencies, and missed values. Descriptive statistics were computed to obtain frequencies and percentages of KPA on WASH. Pie chart and bar graphs were used to present the responses for not having toilets and percentages of respondents for each critical handwashing practices, respectively. Each knowledge, attitude, and practice on WASH and related questions were presented using frequencies and percentages in tables. Confidence intervals (CI) for good practice, good knowledge, and good attitude were calculated.

### 2.4. Ethical Approval and Consent

All procedures performed in this study were in accordance with the ethical standards of the institutional and national research committee and with the 1964 Helsinki Declaration and its later amendments or comparable ethical standards. Ethical clearance and approval was obtained from Tigray Health Research Institute Institutional Review Board by a reference number of 0050/2010, and an official support letter was obtained from Tigray Regional Health Bureau. Oral informed consent was obtained from each study participant.

## 3. Result

### 3.1. Sociodemographic Characteristics of the Study Population

A total of 756 households were included in the study. The response rate was 99.6%, and 574 (75.9%) of the respondents were females. Nearly six out of 10 respondents, 440 (58.2%), were unable to read and write. A total of 350 (46.2%) households had a family size of less than 5. Almost half of the respondents, 393 (52%), had daily income of less than one dollar (Table 4).

### 3.2. Knowledge of the Respondents on WASH

The findings revealed that 741 (98%) of the respondents know the health consequences of improperly managed liquid wastes, and most of them, 710 (93.9%), were using clean water source for washing hands. Nine in ten, 671 (89.2), of the respondents know that latrine is essential and obligatory for every HH. Good knowledge on WASH was observed in 42.2% (95% CI: 38.7%, 45.7%) of the respondents (Table 1).

### 3.3. Attitude of the Respondents on WASH

Based on the findings of the study, 719 (95%), 712 (94%), and 708 (94%) of the respondents agreed that waterborne diseases can be prevented through consumption of safe water, boiling water before consumption helps to remove disease causing microorganisms, and diarrheal diseases are caused by poor personal hygiene and sanitation, respectively. Nine in ten, 683 (91%), of the respondents consider diarrheal diseases are transmittable, and 702 (93%) think washing hands after using latrine prevents diarrheal diseases. Besides, one in four, 186 (25%), of the respondents think that washing hands with water alone is enough to fully sanitize hands, and about 131 (17%) agreed that children's stool is free from disease-causing germs. Positive attitude on WASH was observed in 48.5% (95% CI: 44.9%, 52.0%) of the respondents (Table 2).

### 3.4. Practice of the Respondents on WASH

A total of 680 (90%) HHs were using protected water sources (pump/spring) for domestic uses and only 139 (18.3%) of the HHs treated water at HH level by boiling or using Wuha-Agar/chlorine. Majority, 659 (87%), of the HHs spent less than or equal to 30 minutes to fetch water and the average consumption of water was less than 20 liters per person per day in the majority of the respondents, 668 (88.4%). A total of 700 (92.6%) HHs had inappropriate waste disposal practice. Only 267 (35.3%) of the HHs had latrine, and among the HHs with latrine, only 40 (14.98%) of them had handwashing facility. Among HHs with latrine, 224 (84%) utilized their latrines. Almost all (98%) of the HHs, who do not have latrine, defecate in the open field. Good practice on WASH was observed in 49.2% (95% CI: 45.6%, 52.7%) of the respondents (Table 3).

**Figure 2:**
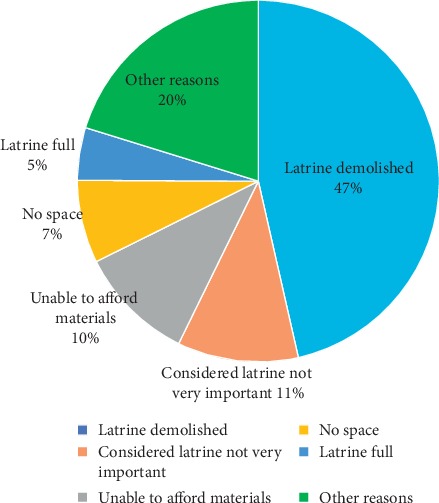
Respondents' reasons for not having latrine, Tigray, 2018, *n* = 756.

The interviewed respondents have indicated that the main reason for not having toilet was latrine demolished due to the traditional nature of the latrines (Figure 2).

**Figure 3:**
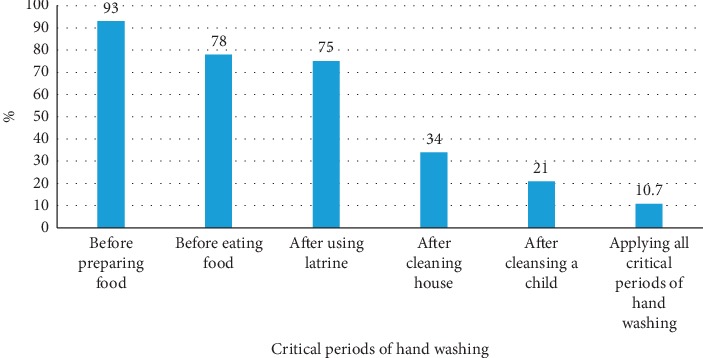
Handwashing practice during critical times, Tigray, 2018, *n* = 756.

Critical hand washing is one of the critical practices of WASH. Responses of the respondents indicate that 93% of them wash their hands before preparing food. The least reported critical handwashing practices were after cleaning a house (34%) and after cleansing a child (21%) (Figure 3).

## 4. Discussion

A cross-sectional study was conducted to assess knowledge, attitude, and practice (KAP) of WASH among rural residents in Tigray region, Northern Ethiopia. Good knowledge, positive attitude, and good practice on WASH were observed in 42.2% (95% CI: 38.7%, 45.7%), 48.5% (95% CI: 44.9%, 52.0%), and 49.2% (95% CI: 45.6%, 52.7%) of the respondents, respectively. Nine in ten of the HHs were using protected water sources for domestic uses, and nearly one in five of the HHs treated water at HH level. Inappropriate waste disposal and housing environment cleanliness were observed in 60%, 93%, and 61% of the HHs. This study has also indicated that only 15% of the 267 HHs with latrine and only 5.3% of the total HHs surveyed had handwashing facility, and only one in ten (10.7%) of the respondents practiced hand washing at critical periods.

Good knowledge on WASH was observed in 42.2% (95% CI: 38.7%, 45.7%) of the respondents. A study in periurban areas in Northwest Ethiopia showed that 75.7% of their respondents had good knowledge [13]. Nine in ten (89.2%) of the respondents know latrine is essential and obligatory for every HH. A similar study in North Ethiopia reported that 94% [14] and 95.2% [13] of respondents know the significance of latrine for health. However, a study in Dire-Tiyara district, Eastern Ethiopia, indicated that 48.3% of the respondents think latrines are only intended for rich people [14]. These differences might be due to the variations in health care promotion services and differences in socioeconomic status.

Positive attitude on WASH was observed in 48.5% (95% CI: 44.9%, 52.0%) of the respondents. A study in periurban areas in Northwest Ethiopia showed that 73.6% of their respondents had positive attitude [13]. Besides, 83% of the respondents have unfavorable attitude towards children's stool, 85% agreed consumption of safe and enough water can prevent waterborne diseases. Another study reported that 91.5% think children's feces may contain germs [13]. Correspondingly, a study in India reported that 95% of the respondents perceive quality of water affects health [15]. These differences might be attributed to variations in quality and coverage of community health care services in the communities.

Good practice on WASH was observed in 49.2% (95% CI: 45.6%, 52.7%) of the respondents. Nine in ten, 680 (90%), of the HHs were using protected water sources for domestic uses, and nearly one in five (18.3%) of the HHs treated water at HH level. A similar study conducted in Northwest Ethiopia indicated that four in five (80%) of HHs used pipe water and one-third (34%) of the respondents practiced HH water treatment [13], and a study conducted in 16 small towns in Ethiopia reported that 79% of the HHs used pipe water [16]. On the other hand, a study conducted in Nepal showed that 53.4% of the total population has access to pipe water [17]. Nearly, nine in ten, 659 (87%), of the HHs spend less than or equal to 30 minutes to fetch water which is similar to a study done in India [15]. The average consumption of water by HHs is less than 20 liters per person per day for nearly nine in ten, 668 (88.4%), of respondents. In line with this, a study in East Shoa Zone, Ethiopia, reported that three in four, 74%, of the HHs consume less than 20 liters per person per day [18]. Behavioral factors are important in determining the uptake and sustainable adoption of water, sanitation, and hygiene technologies and practices. Hence, behavioral variations across the different areas may have an impact on the difference in WASH practices.

Inappropriate waste collection, waste disposal, and housing environment cleanliness were observed in 60%, 93%, and 61% of the HHs. Differently, a study in Accra, Ghana, indicated that inappropriate waste disposal was observed in only 39% of the study HHs [19]. Nearly one in three of the HHs, 267 (35.3%), have latrine. The 2015 National Sanitation Review Report indicated that the coverage of latrine in Tigray region was 61% [11]. This decline could be due to seasonal and temporary nature of the traditional latrines.

This study indicated that latrine utilization among the HHs was only 30%. This is lower compared to a study done in Kersa Woreda, Eastern Ethiopia, which indicated that only 36.4% of the HHs had latrines and almost all were simple unsanitary traditional pits [20]. A study conducted in rural communities of Gulomekeda district of Tigray, Ethiopia, reported 57.3% latrine utilization, which is much higher [21]. The 2011 baseline report of Tigray region on WASH reported similar latrine utilization, 34% [22], and Debre Markos District, Ethiopia, reported higher utilization of latrines, 63% [23]. These variations might be due to cultural and behavioral communication for changes across the different populations.

Only 40 (15%) of the 267 HHs with latrine and only 5.3% of the total HHs surveyed had handwashing facility, and only one in ten, 10.7%, of the respondents practiced hand washing at critical periods. A similar study from rural HHs of Tanzania reported that only 13.2% of the HHs had handwashing facilities outside latrine [24], and another study in Gedeo Zone, South Ethiopia, showed that handwashing practices at critical periods were reported to be 44.2% [25]. This is an indication that there is lack of integration of health extension programs to enhance the sanitation across the health care tire system.

The strengths of the study include that the study was done in several districts and relatively higher sample size with strict supervision at the time of data collection. In addition to that the study has used expert observations to validate the existing practices of WASH in the households. Despite these strengths, the study was not without limitations. The analysis was only descriptions which lack to identify the factors affecting poor knowledge, attitude, and practice on WASH. Likewise, there might be recall bias for some of the variables during the interview time. Issues that can be addressed through qualitative study cannot be addressed in this study.

## 5. Conclusions

Poor knowledge, unfavorable attitude, and poor practice on WASH were common amongst the residents in rural Tigray, Northern Ethiopia. Therefore, the health extension programs at primary health care should be revitalized in a way that can enhance the interventional measures to improve knowledge, attitude, and practice on WASH.

## Figures and Tables

**Table 1:** Knowledge of respondents on WASH in Tigray region, 2018.

Variable	Category	*N* (%)
Can unsafe water cause diarrheal diseases?	Yes	726 (96.9)
	No	23 (3.1)
Can water get contaminated?	Yes	680 (90.9)
	No	68 (9.1)
Was a clean water source used for hand washing?	Yes	710 (93.9)
	No	19 (2.5)
What are the consequences of liquid wastes?	Expose to diseases	741 (98)
	Does not expose to diseases	15 (2)
Does animal dung cause diseases?	Yes	690 (91.3)
	No	66 (8.7)
Have you got information on WASH in the last 6 months?	Yes	464 (71.1)
	No	188 (28.8)
Source of information about WASH?	Health extension workers	577 (88.5)
	Radio/television	139 (21.3)
	Women development army/community	173 (26.5)
What was the information received on WASH about?	Hand hygiene	531 (70.2)
	Water quality	415 (54.9)
	Latrine	549 (72.6)
	Sanitation	347 (45.9)
Is latrine essential and obligatory for every household?	Yes	671 (89.2)
	No	81 (10.8)
Prevention mechanisms for acute watery diarrhea?	Food hygiene	641 (84.8)
	Hand hygiene	537 (71)
	Sanitation	448 (59.3)
	Using latrine	330 (43.7)
What are the consequences of not washing hands?	Expose to various diseases	744 (98.4)
	Does not expose to diseases	12 (1.6)
Knowledge on WASH	Poor	437 (57.8)
	Good	319 (42.2)

**Table 2:** Attitude of respondents on WASH in Tigray region, 2018 (*n* = 756).

Characteristics	Strongly agree, *N* (%)	Agree, *N* (%)	Neutral, *N* (%)	Disagree, *N* (%)	Strongly disagree, *N* (%)
*Attitude on access, quality, and use of water*					
Clean water consumption is important only when one gets sick	27 (4)	56 (7)	20 (3)	379 (50)	274 (36)
Consumption of safe and enough water can prevent waterborne diseases	272 (36)	447 (59)	21 (3)	9 (1)	7 (1)
Defecating near water source can cause contamination	349 (46)	356 (47)	29 (4)	14 (2)	8 (1)
Boiling water before consumption helps to remove disease causing microorganisms	376 (50)	336 (44)	33 (5)	9 (1)	2 (0)
Water containers must always be clean	441 (58)	293 (39)	17 (2)	2 (0)	3 (0)

*Attitude on sanitation and health promotion*					
Cattle dung, if not properly managed, causes health problems	304 (40)	375 (50)	27 (4)	34 (5)	16 (2)
Disposing liquid waste inside the compound does not cause any health problems	32 (4)	96 (13)	28 (4)	376 (50)	224 (30)
Diarrheal diseases are caused by poor personal hygiene and sanitation	329 (44)	379 (50)	29 (4)	9 (1)	10 (1)
Diarrheal diseases are transmittable	389 (52)	294 (39)	40 (5)	19 (3)	14 (2)
Waste can be breeding sites for flies and rodents	401 (53)	320 (42)	17 (2)	9 (1)	9 (1)

*Attitude on latrine access and utilization*					
The significance of latrine is for privacy only	39 (5)	104 (14)	35 (5)	310 (41)	268 (35)
Nonutilization of latrine by neighboring households is unrelated to respondents' family health	31 (4)	121 (16)	85 (11)	342 (45)	177 (23)
Households have obligation to construct their own latrine	410 (54)	300 (40)	27 (4)	8 (1)	11 (2)
Latrine is important for nighttime use only	35 (5)	72 (10)	28 (4)	375 (50)	246 (33)
Latrine structure should include washable slab with super structure and ventilation	21 (3)	8 (1)	22 (3)	354 (47)	338 (45)

*Attitude on hand hygiene*					
Washing hand after using latrine prevents diarrheal diseases	309 (41)	393 (52)	26 (3)	11 (2)	17 (2)
Children's stool is free from disease causing germs	47 (6)	84 (11)	61 (8)	358 (47)	206 (27)
Washing hands with water alone is enough to sanitize hands	28 (4)	158 (21)	55 (7)	363 (48)	152 (20)
Washing hands is more important after eating than before eating food	26 (3)	151 (20)	46 (6)	302 (40)	231 (31)
Hand hygiene and diarrheal diseases are unrelated	32 (4)	63 (8)	56 (7)	438 (58)	167 (22)

*Over all attitude*	*N* (%)				
Unfavorable attitude	388 (51.5)				
Favorable attitude	365 (48.5)				

**Table 3:** Practice of respondents on water, sanitation, and hygiene, in Tigray region, 2018 (*n* = 756).

Variable	Category	*N* (%)
Source of water supply	Protected (pump/spring)	680 (89.9%)
	Unprotected (river/spring)	84 (11.1%)
Time taken to fetch water	≤30 min	659 (87.2%)
	>30 min	91 (12.0%)
Water consumption quantity/person/day	≤10 liters	327 (43.3%)
	10–20 litres	341 (45.1%)
	≥20 liters	88 (11.6%)
Solid waste disposal practice	Appropriate disposal	56 (7.4)
	Inappropriate disposal	700 (92.6)
Have latrine	Yes	267 (35.3)
	No	489 (64.7)
Latrine utilization	Among those having latrine	224 (84)
	Among all households surveyed	224 (30)
Households with latrine have handwashing facility (*n* = 267)	No	225 (85.02)
	Yes	40 (14.98)
Households have handwashing facility (*n* = 756)	No	714 (94.44)
	Yes	42 (5.56)
Material used for hand washing	Water and soap/ash	455 (60.2)
	Water only	301 (39.8)
Respondent clip hand nails regularly	No	115 (15.2)
	Yes	638 (84.4)
Cleanliness of the household compound	Unclean	461 (60.98)
	Clean	295 (39.02)
Household waste collection	Inappropriate	454 (60.05)
	Appropriate	302 (39.95)
Household waste disposal	Inappropriate	703 (92.99)
	Appropriate	53 (7.01)
Practice on WASH	Poor	384 (50.8)
	Good	372 (49.2)

**Table 4:** Demographic and socioeconomic characteristics of respondents in Tigray (*n* = 756).

Variable	Category	*n* (%)
Zone (district)	Central (Adwa)	110 (14.55)
	Eastern (Atsebi Wenberta)	124 (16.4)
	Northwestern (Laelay Adyabo)	121 (16.01)
	Southern (Raya Azebo)	125 (16.53)
	Southeastern (Saharti Samre)	135 (17.86)
	Western (Welkayit)	141 (18.65)

Respondent's role in the household	Mother	527 (69.7)
	Father	137 (18.1)
	Adult daughter/son	91 (12.1)

Sex	Male	182 (24.1)
	Female	574 (75.9)

Age (years)	<30	257 (44)
	30–45	289 (38.2)
	>45	191 (25.3)

Education level	Unable to read and write	440 (58.2)
	Able to read and write	316 (41.7)

Occupational status	Farmer	676 (89.4)
	Merchant	21 (2.8)
	Unemployed	42 (5.4)
	Employed	18 (2.4)

House ownership	Private/personal house	659 (87.2)
	Rental/relative's house	95 (12.6)

Marital status	Married	550 (72.8)
	Single	93 (12.3)
	Divorced	62 (8.2)
	Widow/widower	48 (6.3)

Family size	≤4	350 (46.2)
	>4	401 (53.1)

Property possession	Have radio/television	307 (40.6)
	Have electric line	69 (9.1)
	Have telephone	526 (69.6)

Monthly income in Ethiopian Birr (USD)	<850 (<$1USD/day)	393 (52.0)
	850–1700 ($1-2USD/day)	226 (29.9)
	>1700 (>$2USD/day)	137 (18.1)

## Data Availability

The authors ensure the availability of data and material of this research work, and readers can access the data upon request to the corresponding author.

## Abbreviations

DHS: Demographic and health survey
HHs: Households
KAP: Knowledge, attitude, and practice
WASH: Water, sanitation, and hygiene
WHO: World Health Organization.
